# The role of accreditation in 21st century health professions education: report of an International Consensus Group

**DOI:** 10.1186/s12909-020-02121-5

**Published:** 2020-09-28

**Authors:** Jason R. Frank, Sarah Taber, Marta van Zanten, Fedde Scheele, Danielle Blouin, on behalf of the International Health Professions Accreditation Outcomes Consortium

**Affiliations:** 1 Office of Specialty Education, Royal College of Physicians and Surgeons of Canada, Ottawa, Canada; 2 Department of Emergency Medicine, University of Ottawa, Ottawa, Canada; 3 Foundation for Advancement of International Medical Education and Research, Philadelphia, PA USA; 4 OLVG Teaching Hospital, Amsterdam, The Netherlands; 5 VU Medical Center, School of Medical Sciences, Amsterdam, The Netherlands; 6 Athena Institute for Transdisciplinary Research, Amsterdam, The Netherlands; 7 Department of Emergency Medicine, Queen’s University, Kingston, Canada

**Keywords:** Accreditation, Curriculum, Assessment, Learning environment, Quality improvement, Outcomes

## Abstract

**Background:**

Accreditation is considered an essential ingredient for an effective system of health professions education (HPE) globally. While accreditation systems exist in various forms worldwide, there has been little written about the contemporary enterprise of accreditation and even less about its role in improving health care outcomes.

We set out to 1) identify a global, contemporary definition of accreditation in the health professions, 2) describe the relationship of educational accreditation to health care outcomes, 3) identify important questions and recurring issues in twenty-first century HPE accreditation, and 4) propose a framework of essential ingredients in present-day HPE accreditation.

**Methods:**

We identified health professions accreditation leaders via a literature search and a Google search of HPE institutions, as well as by accessing the networks of other leaders. These leaders were invited to join an international consensus consortium to advance the scholarship and thinking about HPE accreditation. We describe the consensus findings from the International Health Professions Accreditation Outcomes Consortium (IHPAOC).

**Results:**

We define accreditation as *the process of formal evaluation of an educational program, institution, or system against defined standards by an external body for the purposes of quality assurance and enhancement.* In the context of HPE, accreditation is distinct from other forms of program evaluation or research. Accreditation can enhance health care outcomes because of its ability to influence and standardize the quality of training programs, continuously enhance curriculum to align with population needs, and improve learning environments. We describe ten fundamental and recurring elements of accreditation systems commonly found in HPE and provide an overview of five emerging developments in accreditation in the health professions based on the consensus findings.

**Conclusions:**

Accreditation has taken on greater importance in contemporary HPE. These consensus findings provide frameworks of core elements of accreditation systems and both recurring and emerging design issues. HPE scholars, educators, and leaders can build on these frameworks to advance research, development, and operation of high-quality accreditation systems worldwide.

*A strong case is made that the present content, organisation, and delivery of health professionals’ education have failed to serve the needs and interests of patients and populations.*

-Richard Horton, Lancet 2010 [1]

## Background

Effective accreditation is considered an essential ingredient for any system of health professions education (HPE) [2] At the present time, HPE accreditation is undergoing unprecedented scrutiny and change worldwide. While some form of quality monitoring activity is arguably as old as modern medical schools, [3, 4] seminal reports on HPE reform have driven the evolution of accreditation in an accelerating fashion. The Flexner Report (1910) [5] led to the shuttering of dozens of North American medical schools, as well as to changes to curriculum, philosophy of education, and oversight. The 1988 Edinburgh Declaration [6] sought to realign HPE to societal needs and it drove reforms in instructional methods, faculty development, and settings for training. The 2010 Lancet Commission on the Education of Health Professionals for the twenty-first Century [7] demanded the worldwide change to competency-based HPE to better meet the needs of local populations [8]. In 2012, the Accreditation Council for Graduate Medical Education (ACGME) implemented these kinds of changes in American residency education by reorienting US accreditation with graduate outcomes instead of processes [9]. In 2013, the Canadian Residency Accreditation Consortium (CanRAC) also moved to fundamentally overhaul its philosophy, activities, infrastructure, and standards for a more contemporary approach to accreditation [10]. Why has there been all this attention on accreditation reform in recent years? Those highlighting the failings of HPE have advocated for greater attention for accreditation systems. The Lancet Commission, for example, has spotlighted perceptions of ill-prepared graduates in poorly designed HPE systems [7]. The outcomes work of David Asch and others has highlighted unacceptable variations in graduate competence produced by current programs [11–14]. Among the critical reforms of the twenty-first century is a growing call to update HPE accreditation to ensure quality training produces high-quality graduates [9, 15].

Accreditation systems provide oversight and guidance to health professions training programs and articulate a model of quality training to produce practitioners to meet societal health needs [2, 15]. For such a critical function in a professional education system, little has been written to describe the role, elements, impact, and evolution of HPE accreditation. Even less has been done to link accreditation practices to the outcomes of training programs.

## Purpose

To begin a twenty-first century dialogue about the future of accreditation in the health professions, we recognized a need to review the existing literature on accreditation, examine systems worldwide, and develop consensus frameworks on which to build the next generation of accreditation systems. We describe the efforts of an international consensus consortium to 1) identify a global, contemporary definition of accreditation in the health professions, 2) describe the relationship of educational accreditation to health care outcomes, 3) identify important questions and recurring issues in twenty-first century HPE accreditation, and 4) propose a framework of essential ingredients in present-day HPE accreditation.

## Methods

To address these goals, we identified health professions accreditation leaders via a literature search and Google search of HPE institutions, as well as by accessing the networks of other leaders using snowball sampling [16]. We specifically sought out accreditation leaders and scholars from multiple health professions, as well as those with perspectives from across the continuum of a health professional’s career. These leaders were invited to join an international consensus consortium to advance the scholarship and thinking about HPE accreditation—called the International Health Professions Accreditation Outcomes Consortium (IHPAOC, pronounced “epoch”).

We then organized a series of issue identification and consensus activities. We convened the 1st World Summit on Accreditation Outcomes in conjunction with the International Conference on Residency Education in Calgary, Canada in 2013, and a 2nd World Summit in conjunction with the Association for Medical Education in Europe conference in Basel, Switzerland in 2018. At these Summits, we used an iterative group process to identify themes related to the current state of, and future directions for, HPE accreditation. Table 1 lists the breadth of representation at the Summits. We subsequently organized regular (approximately six times per year) international calls and subgroups for the Consortium to further explore the issues identified at the Summit. As part of this process, we developed a series of papers to capture the consensuses formed on the subthemes identified. Each paper was discussed and reviewed by all members of the Consortium.

**Table 1:** Breadth of representation at 2013 and 2018 World Summits

	Total Registrants	Countries Represented	Organizations Represented
**2013**	**82**	**10**	**41**
		United States, Australia, Netherlands, Germany, China, Taiwan, Oman, Canada, Qatar, Barbados	Accreditation Council for Graduate Medical Education, Alberta Health Services, ANZCA, Association of Faculties of Medicines of Canada, Australian Medical Council, Australian National University Medical School, Canadian Medical Association, Catharina Hospital Eindhoven, Children’s Hospital of Eastern Ontario, China Medical University (Taiwan), Collège des médecins du Québec, College of Family Physicians of Canada, College of Physicians & Surgeons of Alberta, Committee on the Accreditation of Canadian Medical Schools, Dalhousie University, Foundation for Advancement of International Medical Education and Research, Hôpital de Montréal pour enfants, Maimonides Infants and Children’s Hospital of Brooklyn, Mayo Clinic, McGill University, McMaster University, Medical Case Center; Karolinska Institutet, Memorial University, Northern Ontario School of Medicine, Oakland University, OLVG Teaching Hospital, Oman Medical Specialty Board, Queen’s University, Ross University, Royal Australasian College of Physicians, Royal College of Physicians and Surgeons of Canada, Sidra Medical and Research Center, The Ottawa Hospital, The Royal Dutch Medical Association, Tom Baker Cancer Centre, University of Alberta, University of British Columbia, University of Calgary, University of Manitoba, University of Saskatchewan, University of Toronto, University of Western Ontario
**2018**	**45**	**15**	**31**
		United States, Australia, United Kingdom, Canada, Oman, Germany, Netherlands, Singapore, Finland, Austria, United Arab Emirates, South Korea, Cameroon, Sudan, France	Accreditation Council for Graduate Medical Education, Association of Faculties of Medicine of Canada, Australian Dental Council, Australian Medical Council, Australian National University, Committee on Accreditation of Canadian Medical Schools, Dieter Scheffner Center for Medical Education, Educational Commission for Foreign Medical Graduates, Foundation for Advancement of International Medical Education and Research, Jhpiego, Johns Hopkins, Korea University, Massachusetts General Hospital, Medical Specialties Council Netherlands, Memorial University, Michigan Medicine, National Cancer Center Singapore, OLVG hospital/VU medical center, Oman Medical Specialty Board, Opportunities In Africa, Pro Medico, Royal College of Physicians and Surgeons of Canada, Sudan Medical Specialization Board, Tan Tock Seng Hospital, The Royal Dutch Medical Association, Ty Dresden, Western Sydney University, United Arab Emirates University, Université Lumière, University of Calgary, University of Plymouth,

In this paper, on behalf of the IHPAOC, we offer an overview of the consensus discussions on the fundamental issues and elements of HPE accreditation.

## Results: contemporary issues in HPE accreditation

### What is accreditation in HPE?

Accreditation can be considered a societal enterprise that is fundamental for both effective HPE and effective health care, but there is no universal agreement on its definition. It has variously been described as a form of quality assurance (QA), an enterprise of continuous quality improvement (CQI), a form of program evaluation, and various combinations of the above. The World Federation for Medical Education (WFME), for example, espouses the following:

*Accreditation is the certification of the suitability of medical education programmes, and of the competence of medical schools in the delivery of medical education* [17].

Similarly, the International Association of Medical Regulatory Authorities uses this statement:

*Accreditation is the process by which a credible, independent body assesses the quality of a medical education program to provide assurance that it produces graduates that are competent to practise safely and effectively under supervision as interns (or equivalent), and have been provided with an appropriate foundation for lifelong learning and further training in any branch of medicine* [18].

The US National Academy of Sciences, Engineering and Medicine defines accreditation in terms of health workforce planning:

*The purpose of accreditation is to build a competent health workforce by ensuring the quality of training taking place within those institutions that have met certain criteria. … Accreditation is a tool for monitoring and ensuring such quality* [15].

Key features that arose in the discussions included the following: the idea that accreditation is a special kind of evaluation targeting a program, an institution, or a system; accreditation involves a comparison against defined standards; accreditation is usually a partnership between the target of the process and some kind of third-party institution; and the view that modern accreditation involves both quality assurance and quality improvement. The IHPAOC members sought a more precise definition of accreditation that both addressed all of these issues and applied to the continuum of HPE. To that end, we developed and adopted the following definition:

*Accreditation in the health professions is the process of formal evaluation of an educational program, institution, or system against defined standards by an external body for the purposes of quality assurance and continuous enhancement.*

IHPAOC also adopted the following goal statement for HPE accreditation:

*Accreditation contributes to ensuring high quality training for a competent workforce prepared to serve societal needs effectively.*

### How does accreditation relate to program evaluation or research in HPE?

Accreditation, program evaluation, and medical education research can sometimes be overlapping endeavours, with shared methods. Table 2 compares these three enterprises.

**Table 2:** Accreditation, program evaluation, and research in health professions education

	Accreditation	Program Evaluation	Research in HPE
Mandate	Ensure safe, high-quality programs, institutions, or systems Often mandated	Accountability to decision makers and improvement	Generate new knowledge
Purpose	Quality assurance and continuous improvement of training	Understanding of context, inputs, implementation, outcomes, impact, or value	Scholarship Advancement of field
Funding	Often government or professional regulatory body	Variable	Variable
Methodologies	A wide variety of quantitative and/or qualitative methods are shared		

Accreditation can be mandated by government or another oversight body or it can be part of a profession’s self-regulation. Meanwhile, program evaluation and research are usually elective activities. Funding for accreditation can be part of a government or regulatory scheme, or it can be from the profession itself. Funding for program evaluation and research come from a wider array of sources, such as grants, endowments, or institutional budgets. In terms of its purpose, accreditation is sometimes seen as a special class of program evaluation, in that programs/curricula/institutions are the evaluands being examined. In the case of accreditation, the evaluation is for the purpose of determining alignment with defined standards from an external body. By contrast, program evaluation and research each employ a vast array of tailored questions, methods, data, and purposes. Accreditation is a special enterprise, with a specific, constrained scope and purpose [19, 20].

### What is the role of accreditation in HPE?

Accreditation’s dual functions of QA and CQI can improve HPE through enhanced training and improved graduate abilities. Table 3 illustrates the spectrum of these QA and CQI perspectives. These two perspectives can often co-exist in many accreditation systems.

**Table 3:** Accreditation as quality assurance and continuous quality improvement

	Quality Assurance	Continuous Quality Improvement
Goal	How can we ensure achievement of minimum standards?	How can we promote excellence and innovation?
Focus	What is below the standard?	What can be done to improve?
Characteristics	Summative	Formative
	Quality judgments	Actionable feedback
	Measurement against predefined requirements and thresholds	Feedback on strengths and areas for improvement
	Preventing harm to learners and patients	Dissemination of innovations, leading practices, and “next” practices
	Culture of episodic, high-stakes evaluation	Culture of continuous enhancement
	Audit model	Coaching model

#### Accreditation as quality assurance

**Fig. 1:**
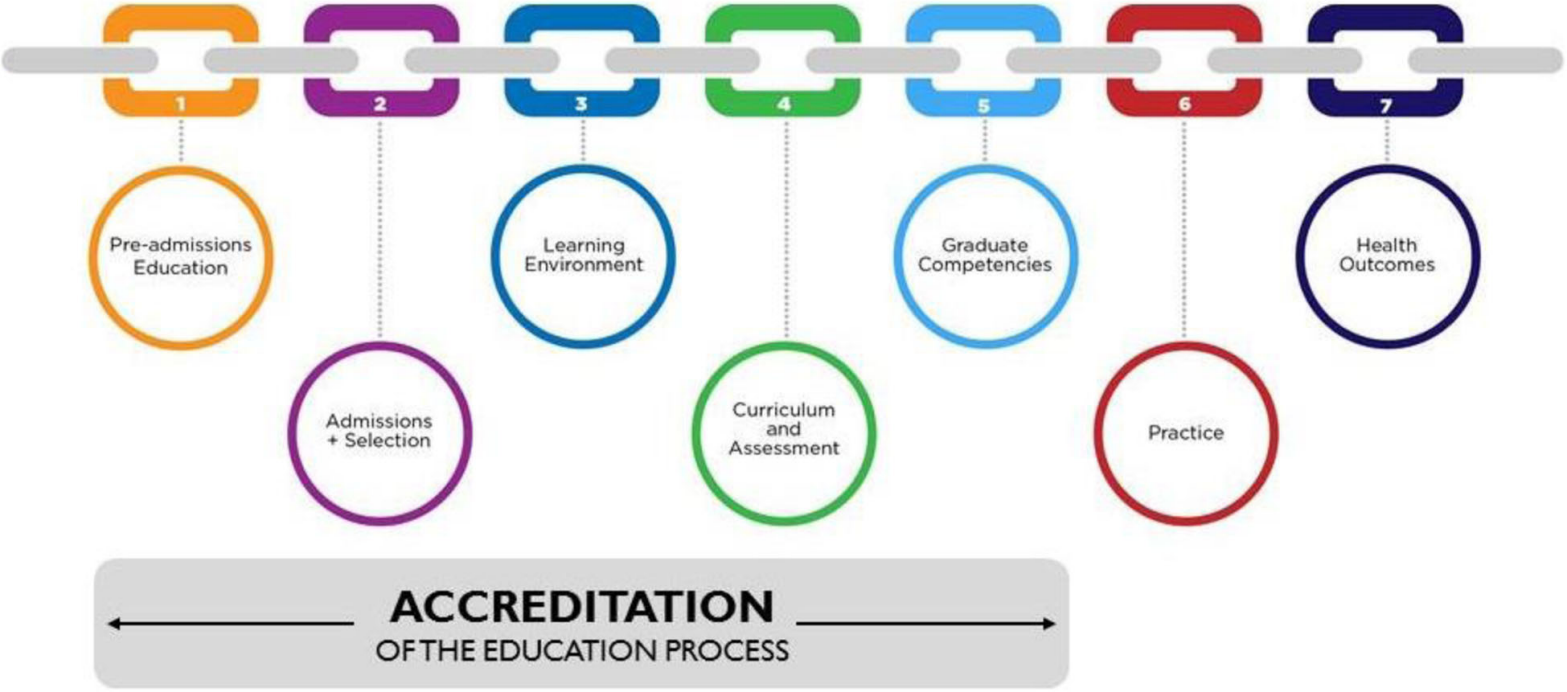
How accreditation connects to the “links in the quality chain” of the health professions

Accreditation is often cited as an essential ingredient in HPE systems: it is a process to ensure that high quality education produces competent graduates to serve a population’s needs. Figure 1 describes how accreditation connects to the “links in the quality chain” of the health professions. In this sense, accreditation is a form of QA in which programs and institutions and/or systems are measured against pre-defined expected characteristics. A decision is made after an accreditation review as to whether the program/institution has met the standard. This mode of accreditation can be high stakes: careers, programs, funding, reputations, services, and learner advancement can all depend on the review’s findings. This is a summative view of accreditation: accreditation as bulwark to quality and sheriff for suboptimal training [2, 15, 17, 18, 21–27].

In its regulatory QA function, accreditation can ensure learning environments are safe and effective for learners. By promoting adherence to minimum standards, accreditation can even prevent negative environments and practises from being perpetuated, preventing harm to learners and patients alike. QA can also focus on minimum thresholds for the quality of curriculum, instructional methods, and assessment practices. Accreditation, in its QA mode, contributes to keeping the profession, its learners, and its patients safe. Accreditation fulfills a kind of professional accountability and helps to ensure public confidence in self-regulated professions [27].

#### Accreditation as continuous quality improvement

Others describe accreditation as truly a formative process of CQI: programs, institutions, or systems are measured against standards to provide feedback on areas of strength and areas that can be improved [28–37]. Collecting accreditation information in program or institutional portfolios or “report cards” can support ongoing monitoring and improvement of key aspects of education [20, 36, 37]. Ever-evolving third-party standards can be deployed to identify and ameliorate weaknesses in HPE institutions and curricula, with the intention of creating a culture of continuous enhancement. In this way, accreditation can prevent the “fossilization” of curriculum (something Abrahamson called “curriculum ossification”) [38]. Accreditation processes, using onsite surveyors and peer review, can also disseminate innovations and good practices. In the mode of accreditation as CQI, society is served by ensuring a continuous quest for the most effective curricula to produce the best possible graduates.

### How does accreditation contribute to health care outcomes?

In creating standards of educational quality, the accreditation process communicates the values of a profession or professional community. The creation of these standards should consider societal needs for the health professions, aligning them with desired health care outcomes [2]. There is clear evidence that the quality of HPE has a powerful impact on graduate outcomes. Work by Asch [11],Tamblyn [39], Bordage [40], and others clearly demonstrates the profound and permanent impact of the educational setting on graduate abilities and practice patterns. Evidence also suggests that there are factors contributing to physician performance variation that occur outside of clinical skills and knowledge acquisition, such as organizational and systemic factors, thereby making the case for a holistic accreditation system to examine these factors in the learning environment [9, 14].

The emerging work of authors such as Braithwaite and colleagues has provided validity evidence for the positive impact of accreditation activities on desired clinical and institutional outcomes [41–43]. In HPE systems, accreditation contributes to a virtual “value chain” through its impact on the quality of training. By ensuring that minimum requirements are met, accreditation can decrease variation in education and practice, and promote adoption of accepted innovations. Accreditation, through both QA and CQI, can influence the quality of learner selection, curriculum content, teaching activities, learning environments, assessment systems, and ultimately the competence and practice of graduates. It is the practice of these graduates that directly impact health care outcomes. Therefore, accreditation is an essential ingredient in an effective health care system [2, 7, 9, 11].

### What are the common core elements of accreditation systems in HPE?

Accreditation systems around the world are numerous and varied. Each has evolved in its unique context and is composed of unique features [44]. IHPAOC identified the need for a common typology of core elements of accreditation systems, and so proposed a simple framework. The 10 core elements are listed in Table 4. Accreditation systems across the continuum of HPE typically have these components, although with great variation. Design features related to these various elements are elaborated in another IHPAOC paper by Taber et al. [45].

**Table 4:** 10 core elements of accreditation systems

Accreditation system element and definition	
1. **Mandate:** The role and purpose of the accrediting body in reviewing the quality of educational programs, institutions, or systems.	
2. **Accreditation standards (Criteria, Requirements):** Measures or generally accepted benchmarks used in making decisions about the quality of a program, institution, or system.	
3. **Application for accreditation:** The process of reviewing an initial request for accreditation by a program seeking to demonstrate compliance with established standards, and which results in a decision about whether to grant new (first-time) accreditation.	
4. **Self-study (self-evaluation, self-assessment):** The internal process of reflection undertaken by a program, institution, or system to evaluate compliance with externally established standards.	
5. **External assessment of standards:** The process of determining the level of compliance of a program, institution, or system with established accreditation standards, undertaken by individuals external to the program, institution, or system.	
6. **Accreditation reports:** The final report by external evaluators regarding the level of compliance of the program, institution, or system with established standards.	
7. **Accreditation decision:** The final decision on accreditation status, and its associated follow-up, as determined by the accrediting body.	
8. **Accreditation cycle:** The phases of an accreditation process dictating how often each program, institution, or system is re-evaluated for compliance with the standards, including the types of phases and activities in the process and any follow-up activities that must occur between external assessments.	
9. **Site review model:** The approach used by the accrediting body in determining the composition of its external site review team, as well as processes for recruiting, assigning, training, and assessing team members.	
10. **Accreditation system administration:** The approaches used by the accrediting body to support the administration and operationalization of the accreditation process; this component includes the business model, the technology used (if any), system review and improvement (including research and scholarship), and oversight and risk management.	

For each of these elements, it should be noted that there is no clear evidence or consensus as to which features are essential for accreditation to contribute to quality outcomes. However, the power of these core elements is in their promise as a kind of lingua franca for accreditation system design and comparison.

### Trends and tensions: emerging developments in HPE accreditation?

As a final task, IHPOAC participants highlighted several trends and tensions as HPE evolves in the unique context of the twenty-first century.

1. *Summative* vs. *formative? QA* vs. *CQI?* The first overarching theme deals with the tension between the QA and CQI functions of accreditation systems. This is a perennial debate; one that continues to this day. While both perspectives on the role of accreditation involve the comparison of educational quality data to a standard, the CQI view goes beyond making a summative judgment based on the identified gaps. CQI accreditation attempts to provide detailed information on how to enhance a program or institution, perhaps even coaching on how to achieve a higher level of quality. This dichotomy is closely related to another debate as to whether accreditation systems should be fundamentally formative (information provided for the express purpose of improvement of the target) or summative (judgment made on the merit or status of a target). IHPAOC members identified that accreditation designers should explicitly identify and communicate the goals of the process, as well as how those goals relate to QA vs. CQI [45].
2. *Continuous* vs. *episodic?* Participants in the Summit identified a shift from episodic, occasional “biopsies” of programs/institutions/systems to more continuous sampling of information on the state of those targets. Once again, the IHPAOC members felt that this issue should be tailored to the purpose of the accreditation system. Episodic sampling tended to be higher stakes and to involve expensive periodic information gathering; however, it allowed low periods between accreditation activities. In some ways, episodic reviews could be less disruptive to educational work. On the other hand, the argument for continuous elements in accreditation processes is that they allow accreditors to monitor changes in HPE over time, intervene early to ensure adherence or improvement, and ensure that conversations about quality and good practices are always present in programs, institutions, and systems. Adding to this discussion, the IHPAOC paper authored by Akdemir et al. uses the seven core values set out in a study of government oversight to examine three medical education systems [46].
3. *Onsite visits* vs. *document reviews?* Another issue—one related to variations in accreditation practices—relates to the methodology of data gathering. Document reviews are efficient ways to gather information on an educational target, comparing what is described to accreditation standards. However, others advocate for more expensive, and resource-intensive, onsite reviews (sometimes called “surveys”) by expert peer or hired reviewers who look for evidence of adherence to standards in the actual educational environment. Onsite reviews have the obvious advantages of first-hand information, contact with multiple participants, and access to richer sources of information [45].
4. *Peer review* vs. *accreditation expert review?* Who should conduct accreditation reviews? IHPAOC members identified a debate between hired experts who are dedicated to accreditation full time and selected peer reviewers who provide authentic perspectives reflective of their own experiences. With no evidence to guide the choice, Summit participants concluded that the decision should be based on philosophy and practicality. For example: Is authenticity or expertise more important? What is the availability of experts vs. peers? Is cross-fertilization of innovation important [46, 47]?
5. *Outcomes* vs. *process measures?* The final tension occurring in debates among accreditors worldwide is related to how much to weight standards to process (e.g., Does this teaching occur?) vs. outcomes (e.g., Can graduates perform this surgery?). Process measures were characterized as surrogates for the desired outcome in some cases, and they are often easier to measure than outcome measures, which can be complex, temporally distant, and confounded. Summit participants identified a distinct shift to greater, but not exclusive, use of outcome measures for accreditation standards as more data become available [48].

## Discussion

HPE is often cited as an essential component of the health care system of any nation. However, there is evidence that HPE suffers from poor outcomes and unacceptable variation in graduate abilities. There is also evidence of patient harm. Accreditation has been identified as a solution to these challenges facing the health professions, one that can promote both adherence to minimum standards and continuous improvement. There is now an evidence base that supports accreditation as “links in a quality chain” (Fig. 1) and measuring educational activities versus standards has a powerful driving affect on HPE effectiveness. Accreditation is essential to the vitality of a profession and ensures graduates are safely and effectively prepared for contemporary practice [2–4, 7, 9, 15, 18, 21, 49].

The founding of the IHPAOC and the launch of the 1st and 2nd World Summits on Accreditation Outcomes provided a unique opportunity to advance practice and knowledge about accreditation as an enterprise within HPE. The consensus network worked to define HPE accreditation, as well as its role in HPE systems. In doing so, we identified early evidence to guide the design and practice of an accreditation enterprise. Accreditation is limited by lack of a large evidence base and challenged by several philosophical and practical debates and controversies, as well as the lack of a common framework of core elements. This global network has now proposed direction for these core elements and the issues facing HPE accreditation as it continues to evolve. There is an urgent need to build on this work to evaluate and innovate on HPE accreditation to enhance training and, thereby, enhance care.

## Conclusion

HPE accreditation plays a fundamental role in the health workforce for the nations of the world. We report the findings of an international consortium on HPE accreditation that educators around the world can build upon to advance the quality of HPE. By adopting a common definition and identifying recurring issues and the taxonomy of elements, we can begin to compare, learn from, and build upon the diversity of HPE accreditation systems worldwide.

## Data Availability

Not applicable.

## Abbreviations

ACGME: Accreditation Council for Graduate Medical Education
CQI: Continuous quality improvement
HPE: Health professions education
IHPAOC: International Health Professions Accreditation Outcomes Consortium
QA: Quality assurance
WFME: World Federation for Medical Education
